# Tissue Engineering and Dental Implantology: Biomaterials, New Technologies, and Stem Cells

**DOI:** 10.1155/2016/5713168

**Published:** 2016-03-17

**Authors:** Gilberto Sammartino, David M. Dohan Ehrenfest, Jamil A. Shibli, Pablo Galindo-Moreno

**Affiliations:** ^1^Department of Oral Surgery, Faculty of Medicine, University of Naples Federico II, Via Pansini 5, Edificio 14, 80131 Naples, Italy; ^2^Department of Oral and Maxillofacial Surgery, University of Michigan Health System, Ann Arbor, MI 48109-1078, USA; ^3^LoB5 Research Unit, School of Dentistry and Research Center for Biomineralization Disorders, Chonnam National University, Gwangju 500-757, Republic of Korea; ^4^Department of Periodontology and Oral Implantology, Dental Research Division, University of Guarulhos, Guarulhos, SP, Brazil; ^5^Oral Surgery and Implant Dentistry Department, School of Dentistry, University of Granada, Granada, Spain

Over the past 2 decades, tissue engineering has emerged as an alternative technique to repair and restore function of damaged or diseased tissues, and this research topic is growing quickly in the clinical fields. Through translational and transdisciplinary research, tissue engineering combines the attributes of biochemical and biomaterial engineering with the aim of creating bioartificial tissues and organs. For the oral and maxillofacial surgeon, the reconstruction of maxillofacial defects in hard and soft tissues is an ongoing challenge; therefore, the new clinical applications of tissue engineering are important endeavors in oral surgery in general and dental implantology and periodontology in particular.

These new techniques are often combined with new digital approaches (digital radiology and treatment planning, optical imprint, CAD-CAM design of materials, etc.) in order to plan complex rehabilitation, to guide surgical steps related to the prosthetic plan, or to design custom-made biomaterials for tissue engineering applications, for example. Digital dentistry is a wide topic regrouping any dental technology or device that incorporates digital or computer-controlled components, in contrast to that of mechanical or electrical components alone. This new aspect of dentistry is growing very fast in the field and is strong support for tissue engineering and dental implantology. It is an exciting time to be in the dental profession as more technologies are being introduced, which make dentistry safer, faster, more enjoyable, and often better as a whole. These digital technologies are rapidly advancing: new tools such as intra/extraoral scanners [1], cone beam computed tomography (CBCT) scanners [2, 3], computer-aided design/computer-aided manufacturing (CAD/CAM) software [4], and innovative fabrication procedures such as 3D printing and layered manufacturing are changing the way we treat our patients [5, 6].

In parallel to this digital evolution, the stem cells experimental development is a fundamental part of tissue engineering research. Recently, for example, human umbilical cord mesenchymal stem cells (hUCMSCs) have been regarded as a promising candidate for tissue regeneration. Furthermore, it has been reported that hUCMSCs can be induced into odontoblast-like cells* in vitro* and* in vivo* [7]. Even the dental pulp stem cells (DPSCs) were explored, due to their rapid proliferation and capability of forming woven bone* in vitro* and compact bone* in vivo*; and studies are searching for the factors that trigger the osteogenic differentiation of DPSCs for their potential use in bone tissue engineering. Many therapeutic protocols using stem cells are daily tested for different pathologies.

If stem cells and digital developments are important, they are only two elements of the wide range of technologies under development in the domain of tissue engineering. Biomaterials are also a major component of tissue engineering, particularly implantable materials and biological agents as a very active field of clinical regenerative medicine. Implantable biomaterials can take numerous forms and their applications constitute a major source of innovation and investigation: new bone materials, new titanium or ceramic implant design and surfaces, new surgical adjuvants such as platelet concentrates, and so forth. The objectives of all these biomaterials are to repair and to restore function of damaged or diseased tissues and sometimes to promote tissue regeneration. In parallel, the understanding and developments of molecular mediators or biologic agents have increased in the last decade, especially in periodontology and dental implantology. For example, biological agents such as recombinant human Platelet-Derived Growth Factor (rhPDGF-BB), Enamel Matrix Derivate (EMD), and Bone Morphogenetic Proteins (BMPs) [8] and various forms of platelet concentrates (Platelet-Rich Plasma (PRP) and Platelet-Rich Fibrin (PRF)) have been used in many clinical situations, with interesting results in periodontal regeneration and bone augmentation procedures [9, 10].

As it was previously stated [11], these research fields are the most active translational research topics in orofacial sciences. Any research about these new implantable materials or techniques requires basic sciences research,* in vitro* and* in vivo*. For example, the understanding and development of Leukocyte- and Platelet-Rich Fibrin (L-PRF) and associated biotechnologies, which are nowadays one of the growing topics for applied clinical regenerative medicine [9, 10, 12], require pharmacologic, biological, and tissue engineering concepts to be tested, validated, optimized, and finally redeveloped for extended applications in other fields [13, 14]. Finally, implantable materials are also good examples of translational research as they require accurate engineering of the chemical and morphological characteristics of the materials [15], their correlation and validation with biological behaviors and concepts, their validation* in vivo* and in humans, and finally the understanding of their long-term clinical outcomes and eventual pathologies, as previously stated [11].

In conclusion, we are now living in the early era of tissue engineering and regenerative medicine, and applications are numerous in dental implantology. New biomaterials and technologies are the key for the development of this field, and their development requires a significant endeavor in translational and multidisciplinary research, to satisfy the needs for clarity, efficiency, and reproducibility of this still pioneer field.
